# Case Report: A Case of Gallbladder Carcinosarcoma With Osteoclast-like Multinucleated Giant Cells that Was Associated With RANK‐RANKL Signaling

**DOI:** 10.3389/pore.2022.1610134

**Published:** 2022-03-23

**Authors:** Ayumi Niwa, Hiroyuki Tomita, Naoki Watanabe, Shunya Kiriyama, Akira Hara, Takuji Tanaka

**Affiliations:** ^1^ Department of Tumor Pathology, Gifu University Graduate School of Medicine, Gifu, Japan; ^2^ Department of Diagnostic Pathology (DDP), Research Center of Diagnostic Pathology (RC-DiP), Gifu Municipal Hospital, Gifu, Japan; ^3^ Department of Surgery, Gifu Municipal Hospital, Gifu, Japan

**Keywords:** case report, gallbladder, carcinosarcoma, osteoclast-like multinucleated giant cell, RANK-RANKL signaling

## Abstract

**Introduction:** Gallbladder carcinosarcoma with osteoclast-like multinucleated giant cells is known to be most uncommon form of gallbladder cancer. Owing to its rarity, the pathogenesis of gallbladder carcinosarcoma with osteoclast-like multinucleated giant cells is largely unknown.

**Case Presentation:** We present a case of carcinosarcoma with osteoclast-like multinucleated giant cells in the gallbladder. A 57-year-old woman visited our hospital due to jaundice. An examination revealed calculous cholecystitis and gallbladder carcinoma. After cholecystectomy, macroscopic examination disclosed one whitish mass and another distinct brown and pendulous mass in the body of the gallbladder. A pathological examination revealed that each mass had a different histological type: adenosquamous carcinoma and carcinosarcoma with osteoclast-like multinucleated giant cells. Immunohistochemistry revealed that these osteoclast-like multinucleated giant cells are CD68(+), CD163(−), and MIB-1(−). In addition, the osteoclast-like multinucleated giant cells showed the strong expression of RANK and sarcoma cells around the osteoclast-like multinucleated giant cells, were positive for RANKL. Furthermore, RUNX2 was positive for some sarcoma cells. The result indicated that osteoclastic and osteoblast-like differentiation occurred in our case.

**Conclusion:** To our knowledge, this is the first case to show the interaction of RANK-RANKL signaling in gallbladder carcinosarcoma with osteoclast-like multinucleated giant cells.

## Introduction

Gallbladder carcinoma is the most common malignant neoplasm of the biliary tract (1). The annual incidence is approximately 1–2 cases per 100,000 population in the United States, whereas the incidence of gallbladder carcinoma is relatively high in Japan (7/1,00,000) (2, 3). Adenocarcinoma is the most common type of gallbladder cancer, while the frequency of gallbladder carcinosarcoma is low. Tanskin et al. (4) reviewed 656 gallbladder cancers and they reported that 11 cases included sarcomatoid component (1.6%).

Gallbladder carcinosarcoma is a tumor that contains both epithelial and mesenchymal tumor cells. There are few report cases because carcinosarcoma is rare; however, our search of the PubMed database revealed more than 90 reports. Gallbladder carcinosarcomas have a poor prognosis and often infiltrate and metastasize to other organs. Zang et al. (5) reviewed 68 cases of gallbladder carcinosarcoma and they reported that the mean survival was 17.5 months and the one-year and five-year survival rates were 19 ± 5% and 16 ± 5%, respectively.

Gallbladder carcinosarcoma itself is rare, gallbladder carcinosarcoma with osteoclast-like multinucleated giant cells is even less common. Taskin et al. (4) reported that among 11 cases of gallbladder carcinosarcoma, only one was accompanied by the appearance of osteoclast-like multinucleated giant cells.

It is known that the differentiation and activation of osteoclastic multinucleated giant cells are induced *via* interaction with receptor activator of nuclear factor-κB (RANK) and RANK-Ligand (RANKL), which are known to be contained in the tumor necrosis factor (TNF) and TNF receptor (TNFR) superfamilies. In bone tissue, osteoblasts secrete RANKL, and RANKL binds to RANK on monocyte/macrophage lineage progenitor cells for osteoclastic differentiation (6). Recently, certain carcinomas were reported to express RANK, suggesting an association between RANK-RANKL signaling and tumor development (7). In addition, the RANKL expression of malignant neoplastic cells with osteoclast-like multinucleated giant cells was reported by several authors (8–10). In gallbladder neoplasms including gallbladder cancer and carcinosarcoma, the involvement of RANK-RANKL signaling in oncogenesis is still unknown.

Runt-related transcription factor 2 (RUNX2) is known to be a key transcription factor for differentiation and maturation process of osteoblasts from mesenchymal stem cells (11). It was reported that RANKL bound to RANK on the surface of osteoblasts and promoted the expression of downstream RUNX2 for osteoblast differentiation and maturation (12). RUNX2, which is related to RANK-RANKL signaling, is also poorly unknown for the association with gallbladder cancer.

We herein report a rare case of gallbladder carcinosarcoma and adenosquamous carcinoma. The former tumor consisted of spindle cell sarcoma with osteoclast-like multinucleated giant cells and adenocarcinoma. We further analyzed the expression of RANK, RANKL and RUNX2 by immunohistochemistry to investigate the role of RANK-RANKL signaling in this rare gallbladder neoplasm.

## Case Presentation

### Clinical History

A 57-year-old woman with a history of anemia and cataracts, noticed general malaise when taking Kampo medicine for approximately 3 weeks prior to her admission. Apart from this symptom, she had been healthy all her life and was a non-smoker with no history of alcohol consumption. She noticed yellow skin and her family doctor pointed out hyperbilirubinemia of her conjunctiva. She was then admitted to the Department of Gastroenterology of Gifu Municipal Hospital and was hospitalized on the same day in order to clarify the cause and treatment.

Laboratory analyses on admission showed significantly elevated serum levels of CA19-9 (1,080.7 U/ml, reference value: 0–37 U/ml), ALP (818 IU/L, reference value: 106–32 U/L), and γ-GT (325 IU/L, reference value: 9–32 U/L). Her serum total bilirubin level was also elevated (4.9 mg/dl, reference value: 0.5–1.4 mg/ml). Abdominal contrast-enhanced computer tomography (CT) revealed swollen gallbladder with calculous cholecystitis and broadly rooted elevated lesions, suggesting carcinoma. After the removal of gallstones by endoscopic retrograde cholangiopancreatography, the patient underwent extended cholecystectomy.

**FIGURE 1:**
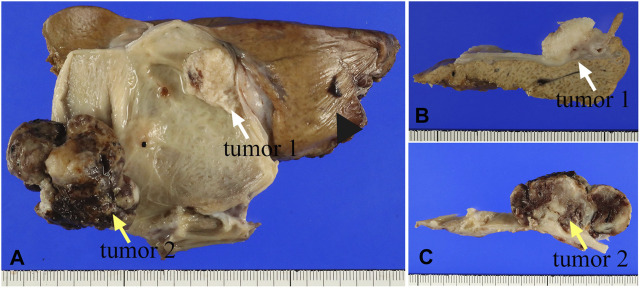
**(A)** The gross appearance of two masses in the resected gallbladder specimen. The white arrow indicates the whitish tumor (tumor 1), while the yellow arrow indicates a larger tumor (tumor 2). The arrowhead indicates the liver. **(B)** The cross-section shows whitish solid mass that was indicated by the white arrow in **(A)**. **(C)** The black to brownish solid mass was indicated by the yellow arrow in **(A)**.

Two solid tumors were observed in the body region of the resected gallbladder (Figure 1A). Macroscopically, they were a whitish solid tumor (3.0 cm × 2.0 cm × 1.0 cm, tumor 1, Figure 1B) invading the muscularis propria (pT1bN0M0) and a black to brownish semi-pedunculated tumor (4.5 cm × 3.5 cm × 2.5 cm, tumor 2, Figure 1C) invading the mucosal layer (pT1aN0M0). The distance between tumors 1 and 2 was more than 1 cm.

The patient did not receive additional treatment such as chemotherapy after the operation, and no recurrence of the tumor was observed even at 6 months.

### Pathological Diagnosis

**FIGURE 2:**
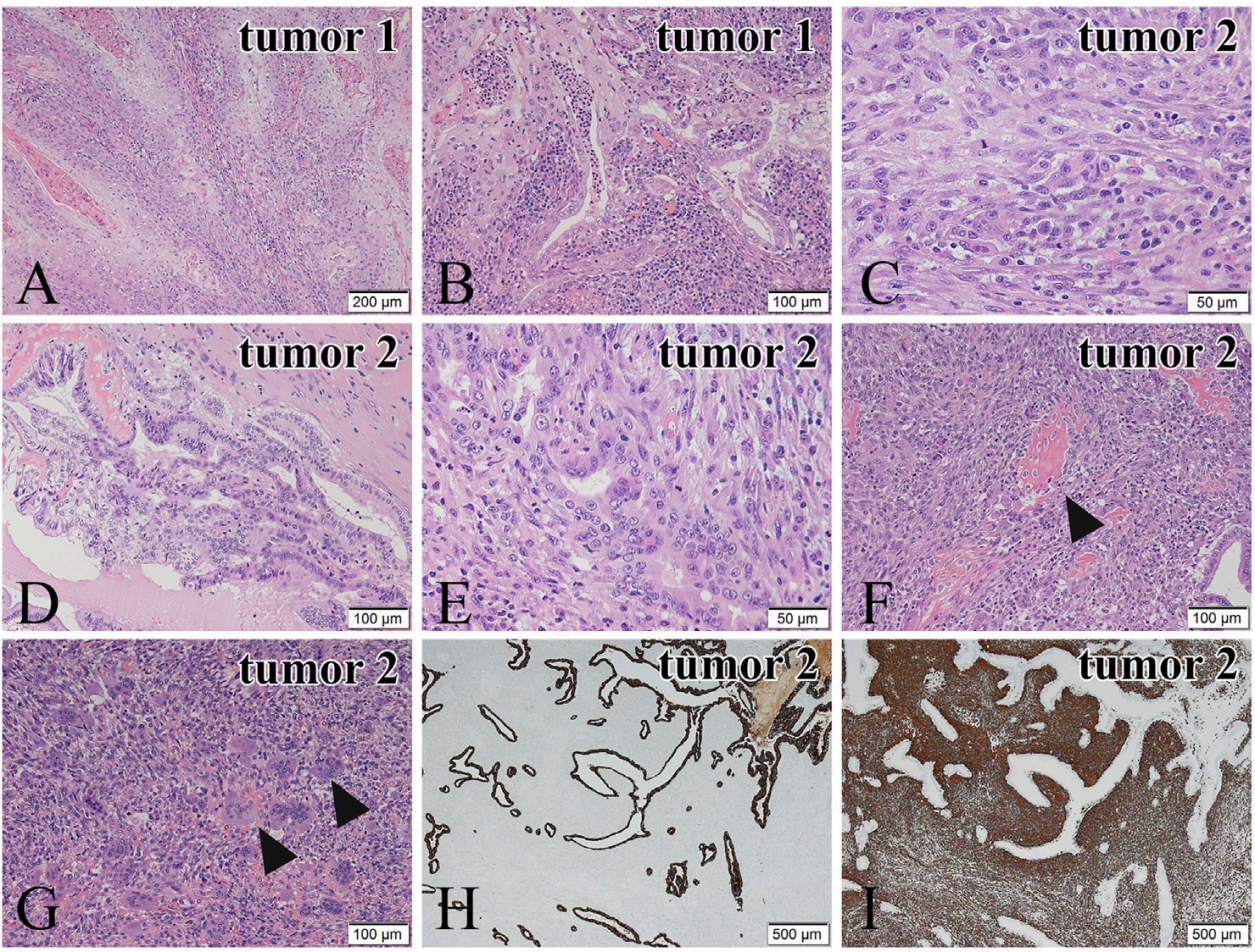
Microscopic features of the tumor 1 **(A,B)** and the tumor 2 **(C–G)**. Immunohistochemical staining of the tumor 2 **(H,I)**. **(A)** In the tumor 1, tumor cells show squamous cell differentiation. **(B)** Transition of the squamous cell carcinoma is noted. **(C)** In the tumor 2, spindle or round tumor cells proliferate solidly. **(D)** Adenocarcinoma cells obtained from the tumor 2 show papillary and tubular growth. **(E)** Transition of adenocarcinoma cells and sarcomatous tumor cells is observed. **(F)** The carcinosarcomatous lesion from the tumor 2. The sarcomatous tumor cells show osteoid production (black arrowheads). **(G)** Many osteoclast-like multinucleated giant cells are present in the sarcoma component of the carcinosarcoma (black arrowheads). **(H)** CK AE1/AE3 immunostaining of the adenocarcinoma component is positive. **(I)** Vimentin immunostaining of the sarcoma component is positive. Hematoxylin and eosin **(H,E)** staining **(A–G)**. Bars in **(A)**, 200 μm; and Bars in **(B–G)**, 100 μm; and Bars in **(C)**, **(E)**, 50 μm; and Bars in **(H)** and **(I)**, 500 μm.

A histopathological examination revealed that the two tumors have quite different microscopic features. The tumor 1 (Figure 1B) consisted of tumor cells with eosinophilic cytoplasm showing stratified squamous epithelial differentiation with keratinization, suggesting squamous cell carcinoma (Figure 2A). In addition, carcinoma cells with glandular structures (Figure 2B) were observed together with the squamous cell carcinoma component. The transition of two components was also noted (Figure 2B). Based on these findings, we histopathologically diagnosed that the tumor 1 was adenosquamous carcinoma.

In the tumor 2 (Figure 1C), atypical spindle and short spindle-shaped, and ovoid tumor cells with round or large distorted nuclei proliferated solidly (Figure 2C), suggesting spindle cell sarcoma. There were 20 mitoses/10HPF of the tumor cells in the tumor 2. An adenocarcinoma component showing tubular and papillary structures (Figures 2D,E), occupying 30% of the area of the tumor 2, was found in the spindle cell sarcoma. We also noted the transition of sarcoma and carcinoma components (Figure 2E). In some parts, osteoid production (Figure 2F) and a large number of osteoclast-like multinucleated giant cells (Figure 2G) were observed in the sarcoma component. Osteoid production was microscopically observed in about 10% of the tumor 2. Osteoclast-like multinucleated giant cells had 4–30 small nuclei without atypia and mitoses. These findings led us to diagnose the tumor 2 as carcinosarcoma with osteoclast-like multinucleated giant cells. Additionally, tumor 2 was an early-stage cancer, so there was no perineural or lymphovascular invasion.

The intervening gallbladder mucosal epithelium between the two tumors was shedding partially and atrophic, and the gallbladder wall was thickened with mild inflammatory cell infiltration and fibrosis, indicating chronic cholecystitis. The dysplasia of gallbladder epithelium in the borders of the carcinosarcoma was observed, and it is a very localized area.

Immunohistochemistry was performed to confirm our histopathological diagnosis. In the tumor 1, the squamous cell carcinoma component was positive for the expression of CK5/6 and p63, whereas the adenocarcinoma component showed negative for the expression of both antibodies. In the tumor 2, the adenocarcinoma component was positive for the expression of CKAE1/AE3 (Figure 2H), EMA and CAM5.2, and the spindle cell sarcoma component was negative for these antibodies. The spindle cell sarcoma component showed positive for the expression of Vimentin (Figure 2I). These results supported our histopathological diagnosis. In addition, the spindle cell sarcoma component was weakly positive for α-SMA and negative for desmin. The MIB-1-positive index of the spindle cell sarcoma component (50%) was higher than that of the adenocarcinoma component (30%). In the area of osteoid production, SATB2 positive cells surrounded the osteoid components (Supplementary Figure S1A), and they could be considered spindle cell osteosarcoma.

### Immunohistochemical Analysis for Osteoclast-Like Multinucleated Giant Cells

**FIGURE 3:**
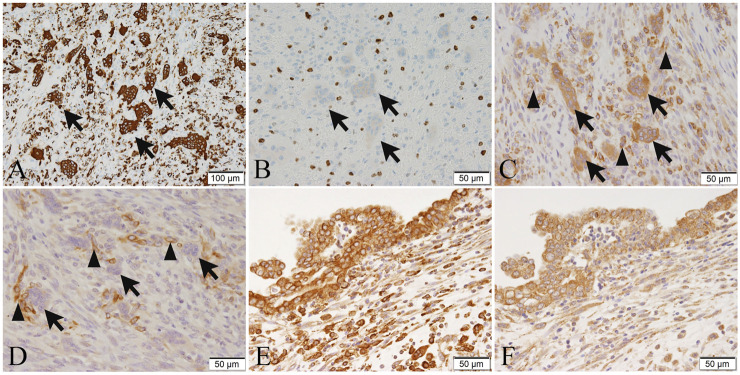
The immunohistochemical expression of CD68 **(A)**, MIB-1 **(B)**, RANK **(C,E)**, and RANKL **(D,F)** in tumor cells or osteoclast-like multinucleated giant cells of the tumor 2. **(A)** Osteoclast-like multinucleated giant cells (arrows) are strongly positive for CD68. **(B)** The MIB-1-positive index in spindle cell sarcoma is 50%. Osteoclast-like multinucleated giant cells (arrows) are negative for MIB-1, suggesting no proliferative activity. **(C)** Osteoclast-like multinucleated giant cells (arrows) are strongly positive for RANK, and some short spindle-shaped and ovoid sarcoma cells (arrowheads) are positive for RANK. **(D)** Osteoclast-like multinucleated giant cells (arrows) are negative for RANKL, while some short spindle-shaped and ovoid sarcoma cells around the osteoclast-like multinucleated giant cells (arrowheads) are positive for RANKL. **(E)** Adenocarcinoma cells are strongly positive for RANK. **(F)** Adenocarcinoma cells are positive for RANKL. Bar in **(A)**, 100 μm; and Bars in **(B–F)**, 50 μm.

Further, to determine the property of the osteoclast-like multinucleated giant cells present in the carcinosarcoma, we performed an immunohistochemical analysis. The osteoclast-like multinucleated giant cells were CD68-positive (Figure 3A) and CD163-negative. The nuclei were partially positive for TP53. MIB-1 staining was negative (Figure 3B), suggesting no proliferative activity and histiocyte-like nature.

Additionally, to investigate the association of carcinosarcoma with osteoclast-like multinucleated giant cells and RANK-RANKL signaling, we performed immunohistochemistry with anti-RANK and anti-RANKL antibodies (sources and dilutions of the antibodies are shown in Supplementary Table S1). The osteoclast-like multinucleated giant cells of tumor 2 were strongly positive for RANK (Figure 3C) but were negative for RANKL (Figure 3D). Some short spindle-shaped and ovoid sarcoma cells were RANK-positive (Figure 3C) and RANKL-positive (Figure 3D). RANKL-positive cells are predominantly located around the osteoclast-like giant cells, and the number of giant cells is also higher in areas with strong RANKL positivity. To reveal this objectively, we counted in five high-power fields (HPFs) and averaged positive cells. Approximately eight cells/HPF (high-power field) are found in areas with strong RANKL positivity. In comparison, only about four cells/HPF areas with weak RANKL positivity.

On the other hand, the spindle-shaped tumor cells were weakly positive to negative for RANK (Figure 3C) and RANKL (Figure 3D). The adenosquamous carcinoma component of tumor 1 was weakly positive or negative for RANK and RANKL. In comparison to spindle-shaped tumor cells and adenosquamous cell carcinoma components, immunohistochemistry revealed that the RANK and RANKL expression of the adenocarcinoma cells was relatively strong (Figures 3E,F).

In our case, osteoid production was observed, and there was no difference in the number of RANK or RANKL positive cells in the osteoid-containing regions.

We have added immunostaining for RUNX2 and evaluated the staining (Supplementary Figure S1B,C). RUNX2 is weakly positive for adenocarcinoma components and sporadically positive for some sarcoma cells.

## Discussion

In this report, we presented the rare case of synchronous double tumors of gallbladder; adenosquamous cell carcinoma and carcinosarcoma with osteoclast-like multinucleated giant cells.

Because of its rarity, the pathogenesis and biological features of gallbladder carcinosarcomas with osteoclast-like multinucleated giant cells remain largely unknown. Although adenocarcinoma developing in the pancreas and gallbladder has similar morphology, osteoclast-like multinucleated giant cells generally appear in the pancreas but not in the gallbladder, when sarcomatoid change occurs (4).

Carcinosarcoma of the gallbladder is called “undifferentiated carcinoma”, “sarcomatoid carcinoma,” or “spindle-cell carcinoma.” Thus, the terminology has not yet been unified(13). The pathogenesis of gallbladder carcinosarcoma remains poorly known. Five theories have been proposed regarding the development of gallbladder carcinosarcoma: (A) mesenchymal components are represented a reactive process, (B) mesenchymal components are considered as true sarcoma (including the collision tumor), (C) mesenchymal components are originated from epithelial components with metaplastic changes, (D) they are originated from embryonic rest, and (E) they are originated from totipotential stem cells(14). Immunohistochemical search favors the (C) theory the most (15, 16). In our case, the dysplasia of gallbladder epithelium in the borders of the carcinosarcoma was observed, and it suggests the origin of the carcinosarcoma.

In our case, not only carcinosarcoma but also adenosquamous cell carcinoma was found in the gallbladder. The most popular theory of the histogenesis of squamous cell and adenosquamous carcinoma in gallbladder carcinoma is that squamous cells arise from squamous metaplasia or squamous differentiation of pre-existing adenocarcinoma(17, 18). Therefore, we suggest that tumor 1 and tumor 2 may have derived separately and independently from pre-existing epithelial components.

Gallbladder carcinosarcoma itself is a disease with a poor prognosis, but it is unclear how osteoclast-like multinucleated giant cells affect the growth and prognosis of carcinosarcoma. To the best of our knowledge, there have been about seven previous reports of gallbladder carcinoma with osteoclast-like multinucleated giant cells. Several histologic types have been described, including adenosquamous carcinoma (19, 20), anaplastic spindle and giant cell carcinoma (21), and undifferentiated carcinoma (22, 23), including carcinosarcoma (Table 1). The depth of the tumor invasion was found to be limited to the muscularis propria in one case, while others invaded other organs (liver, transverse colon, duodenum, etc.,). As for postoperative survival, most patients died within 2 months after surgery, but in one case, a patient survived for 6 years without tumor recurrence. According to the past cases, it is seemed that there is a tendency for the gallbladder carcinoma with osteoclast-like multinucleated giant cells to be advanced tumors. In addition, the origin and cause of proliferation of osteoclast-like multinucleated giant cells is under debate, including whether the phenomenon is associated with gallstones or malignancy.

**TABLE 1:** Histological and clinical findings of previous reports of gallbladder carcinoma with osteoclast-like giant cells.

case (References)	Age	Sex	Histologic type	Tumor invasion	Postoperative survival
Grosso et al. (11)	74	F	Adenosquamous carcinoma	Liver, Transverse colon	2 months
Akatsu et al. (12)	72	F	Adenosquamous carcinoma	Liver	6 years
Manouras et al. (14)	84	M	Undifferentiated carcinoma	Muscularis propria	2 months
Xiao et al. (15)	56	M	Undifferentiated carcinoma	Hilar bile duct, Liver, Duodenum	2 months

Some studies have reported that malignant tumors in other organs with osteoclast-like multinucleated giant cells showed hypervascularity and/or hemorrhage in tumor stroma (6, 24, 25). In our case, the brownish appearance of the gallbladder carcinosarcoma was likely associated with extensive hemorrhage in the stroma. In addition, in certain malignancies (e.g., differentiated hepatocellular carcinoma, uterine leiomyosarcoma, and breast carcinoma) the presence of osteoclast-like multinucleated giant cells has been associated with an aggressive clinical course (6, 26, 27). Hatano et al. (28) reported that osteoclast-like multinucleated giant cells produce vascular endothelial growth factor (VEGF)-C, which promotes tumor growth and lymphangiogenesis.

In several reports regarding gallbladder carcinoma, immunohistochemical analysis was performed for osteoclast-like multinucleated giant cells (4, 21-23). Such cells have been reported to be CD163-negative and CD68-positive (21, 22). CD163 is known to be a specific marker of histiocytes and macrophages (21, 29). In our case, osteoclast-like multinucleated giant cells expressing CD68 had no nuclear atypia or proliferative activity, and we therefore considered that they were mobilized by tumor cells and derived from histiocytes and probably mononuclear macrophages.

In this case, the osteoclast-like multinucleated giant cells were RANK-positive, and the strong expression of RANKL was observed in some ovoid sarcoma cells. In addition, RUNX2 was also positive in some sarcoma cells. These results suggest that osteoblast-like differentiation may occur as shown in normal bone formation.

Malignant tumors with osteoclast-like multinucleated giant cells are also found in various tissues, and RANKL-positive tumor cells have been noted in several neoplasms that develop in the soft tissue, uterus, bladder, and breast (e.g., leiomyosarcoma), as well as sarcomatoid hepatocellular carcinoma (6, 8, 10, 25). In this case, the expression of RANKL was recognized in tumor cells, especially those around osteoclast-like multinucleated giant cells. Denosumab, a widely used anti-RANKL antibody, reduces tumor in patients with giant-cell tumor of bone (30). However, the therapeutic effect on malignant tumors like our case is unknown. Further studies are needed.

To our knowledge, the expression of RANK and RANKL in gallbladder malignancy was analyzed for the first time, as described. Although the accumulation of further cases is necessary, our case gives new insight regarding RANK-RANKL signaling, which may contribute information about the nature and characteristics of the tumor.

## Data Availability

The original contributions presented in the study are included in the article/Supplementary Material, further inquiries can be directed to the corresponding author.

## Supplementary Material

Supplementary Figure S1 The immunohistochemical expression of SATB2 **(A)**, RUNX2 **(B,C)** in tumor cells of the tumor 2. **(A)** In the area of osteoid production, SATB2 positive cells surrounded the osteoid components. **(B)** Some sarcoma cells were positive for RUNX2. **(C)** Adenocarcinoma component was weakly positive for RUNX2. Bars in **(A,C)**, 100 μm; and Bar in **(B)**, 50 μm.
