# Supplementary material for: Case Report: A Case of Gallbladder Carcinosarcoma With Osteoclast-like Multinucleated Giant Cells that Was Associated With RANK‐RANKL Signaling
Source: Pathol Oncol Res. 2022 Mar 23;28:1610134. doi: 10.3389/pore.2022.1610134 (PMC8983827; doi:10.3389/pore.2022.1610134)
Supplement: Supplementary file 1 [file Table1.DOCX]

**Table.S1** Antibodies used in this report

| *Antibody* | *Animal* | *Clonality* | *Dilution* | *Catalog number* | *Vendor* |
| --- | --- | --- | --- | --- | --- |
| RANK | Mouse | Monoclonal | 1:100 | ab13918 | Abcam PLC, Cambridge, UK |
| RANKL | Rabbit | Polyclonal | 1:500 | ab9957 | Abcam PLC, Cambridge, UK |
| SATB2 | Rabbit | Monoclonal | 1:600 | #39229 | Cell Signaling Technology, Inc., Massachusetts, USA |
| RUNX2 | Rabbit | Polyclonal | 1:200 | 20700-1-AP | Proteintech Group Inc., Rosemont, IL, USA |
