# Supplementary figures and images for: Case Report: A Case of Gallbladder Carcinosarcoma With Osteoclast-like Multinucleated Giant Cells that Was Associated With RANK‐RANKL Signaling
Source: Pathol Oncol Res. 2022 Mar 23;28:1610134. doi: 10.3389/pore.2022.1610134 (PMC8983827; doi:10.3389/pore.2022.1610134)

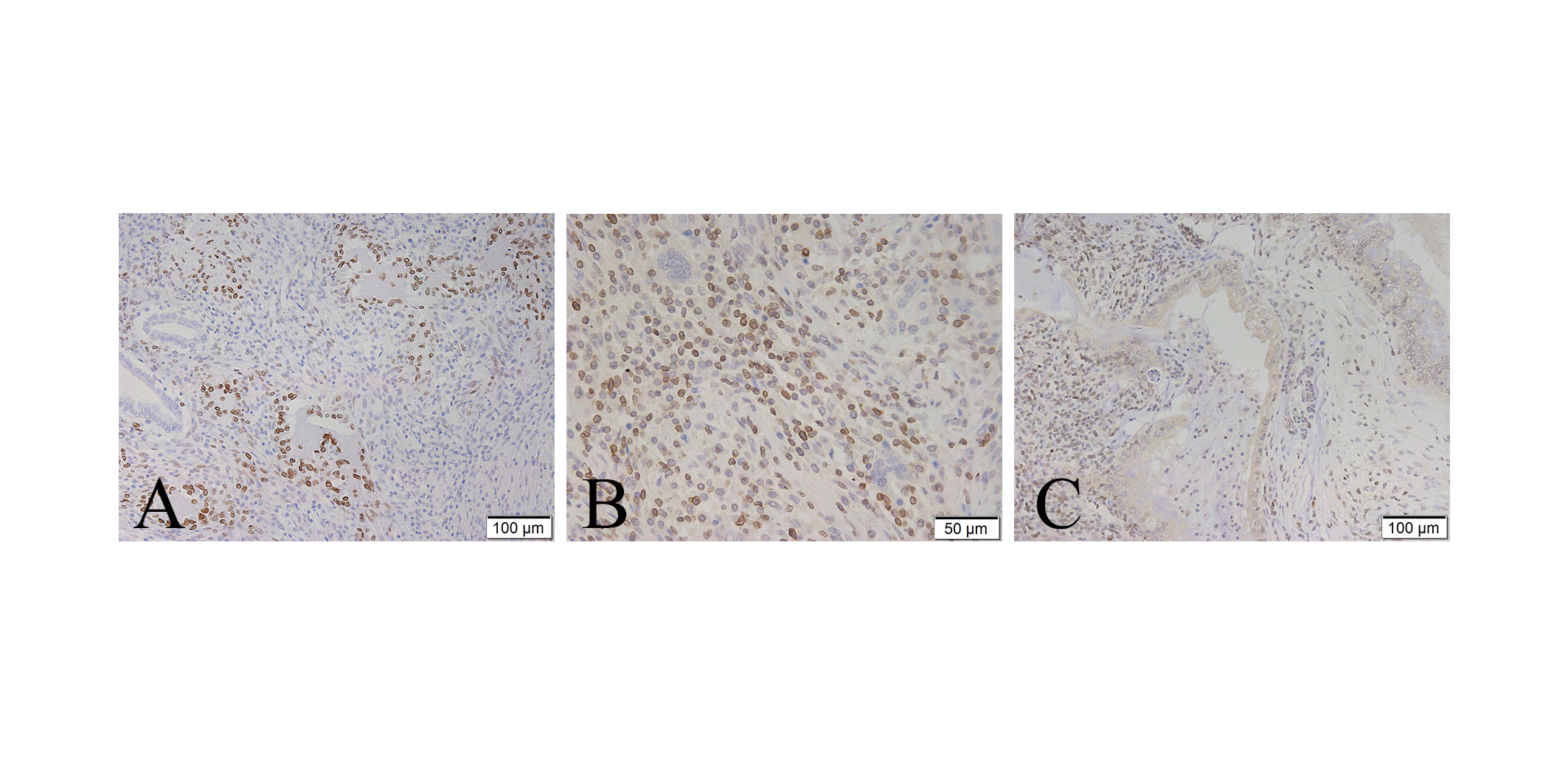

Supplement: Supplementary file 2 [file Image1.JPEG]
